# Denosumab Related Osteonecrosis of the Jaw with Spontaneous Necrosis of the Soft Palate: Report of a Life Threatening Case

**DOI:** 10.1155/2016/5070187

**Published:** 2016-08-28

**Authors:** Mohammed Qaisi, Jamie Hargett, Matthew Loeb, Jeffrey Brown, Ronald Caloss

**Affiliations:** ^1^Oral-Head & Neck Oncology/Microvascular Surgery, Division of Oral & Maxillofacial Surgery and Division of Otolaryngology, John H. Stroger, Jr. Hospital of Cook County, 1900 W. Polk Street, Suite 612, Chicago, IL 60611, USA; ^2^University of Mississippi Medical Center, 2500 North State Street, Jackson, MS 39216, USA; ^3^Advanced General Dentistry, University of Mississippi Medical Center, 2500 North State Street, Jackson, MS 39216, USA; ^4^Department of Oral & Maxillofacial Surgery, University of Mississippi Medical Center, 2500 North State Street, Jackson, MS 39216, USA; ^5^Department of Oral-Maxillofacial Surgery & Pathology, University of Mississippi Medical Center, 2500 North State Street, Jackson, MS 39216, USA

## Abstract

Bisphosphonates have been used for years in the treatment of patients with distant bony metastasis and in the prevention of osteoporosis. One of main side effects of these medications is the development of bisphosphonate related osteonecrosis of the jaw (BRONJ) in a small subset of patients. A new class of medications with a shorter half-life, known as receptor activator of nuclear factor kappa-B ligand (RANKL) inhibitors, was introduced with the hopes of avoiding this side effect. However, reports of osteonecrosis of the jaw after the use of RANKL inhibitors have also been documented. We report on a patient who developed a life threatening osteonecrosis of the jaw with sepsis shortly after switching from a bisphosphonate to a RANKL inhibitor for osteoporosis treatment. This patient developed several soft tissue defects including spontaneous necrosis of the soft palate. To our knowledge this is the first time this presentation has been described.

## 1. Introduction

Bisphosphonate (BP) medications have significantly improved the quality of life of patients suffering from skeletal metastases such as in the cases of prostate cancer, breast cancer, and multiple myeloma [1]. BPs are also used in lower doses for the treatment of osteoporosis, with both oral and intravenous formulations available [1–3]. These drugs have been prescribed for more than 40 years and the pharmacokinetics have become better understood with time [4]. They have a large affinity for the skeleton and have shown preferential binding in bones, which seems to contribute to their very slow rate of elimination from the body, often persisting in the bone several years after discontinuation of BP therapy [5]. One major adverse side effect of prolonged usage of BPs is a well-documented phenomenon known as bisphosphonate related osteonecrosis of the jaw (BRONJ). A diagnosis of BRONJ is made when an area of exposed necrotic bone persists longer than eight weeks in patients with a current or previous history of BP use without a history of radiation therapy [1, 5, 6]. This complication is triggered by invasive dental procedures such as extractions in 75–86% of cases. The incidence of BRONJ is reported to be around 0.7–6.7% for patients being treated for cancer and 0.04–0.2% for patients being treated for osteoporosis [1, 7, 8]. While the exact mechanism by which BRONJ occurs is still not fully understood, it seems that the effect BPs have on osteoclasts and the rate of bone turnover and remodeling is responsible.

In June 2010, a new class of medications known as receptor activator of nuclear factor kappa-B ligand (RANKL) inhibitors, specifically Denosumab, was approved by the FDA for treatment of osteoporosis (Prolia) and bony metastases from solid tumors such as breast and prostate cancer (Xgeva). Denosumab is a human monoclonal antibody that binds to and inhibits the cytokine RANKL, which is an essential mediator in the formation, function, and survival of osteoclasts [6, 9, 10]. This exerts a potent antiresorptive effect which is helpful in reducing skeletal related events (SREs) in cancer and osteoporosis patients. Due to the shorter half-life and lack of covalent binding to bone, it was hoped that Denosumab would provide a similar therapeutic effect to BPs while improving the side-effect profile and preventing cases of osteonecrosis of the jaw (ONJ) [4, 11]. However, in 2010, several reports emerged describing the occurrence of ONJ in patients being treated with Denosumab [6, 12–14].

In this report, we present a patient who developed an advanced case of medication related osteonecrosis of the jaw (MRONJ) shortly after switching from BPs to Denosumab for the treatment of osteoporosis. This patient went on to develop life threatening sepsis and an unexplained soft tissue defect in her soft palate. To our knowledge this is the first time this presentation has been reported.

## 2. Case Report

A 65-year-old Caucasian female with a past medical history of hypertension, gastroesophageal reflux disease, iron deficiency anemia, and rheumatoid arthritis was referred for exposed left mandibular bone and a persistent neck fistula 3 weeks after extraction of tooth #20. She had a concomitant extraoral incision and drainage for a presumed submandibular abscess by an outside practitioner. The patient reported a history of being on bisphosphonates for osteoporosis. She was on Risedronate (Actonel) for a total of 4 years and was then switched to yearly Zoledronic Acid (Reclast) injections for a period of two years with the last dose being roughly 1 year prior to the extraction. She was however started on Denosumab (Prolia) subcutaneously roughly 1 week prior to her extraction.

On physical exam, the patient had a 1 cm cutaneous fistula in left submandibular region. Intraorally there was an area of exposed bone roughly 1 cm in size on the buccal aspect of the mandible in the area of tooth #20; there was a separate area of exposed bone on the lingual aspect. There was minimal amount of thin purulent drainage intraorally and on the neck bandage. A diagnosis of MRONJ was made based on her clinical presentation, and she was scheduled for follow-up with imaging to discuss her treatment options. At follow-up 10 days later, the patient's lesions had increased in size and the two areas of exposed bone were confluent. There was another 5 mm area of exposed bone in the symphysis region and another 5 mm area in the right retromolar pad area. The neck fistula slightly increased in size. CT scan showed sclerotic changes involving the mandible diffusely. Given the diffuse nature of her bony involvement and continued progression of bony exposure, we have elected to watch and wait and allow the necrotic bone to declare itself prior to proceeding with surgical resection and microvascular reconstruction.

**Figure 1:**
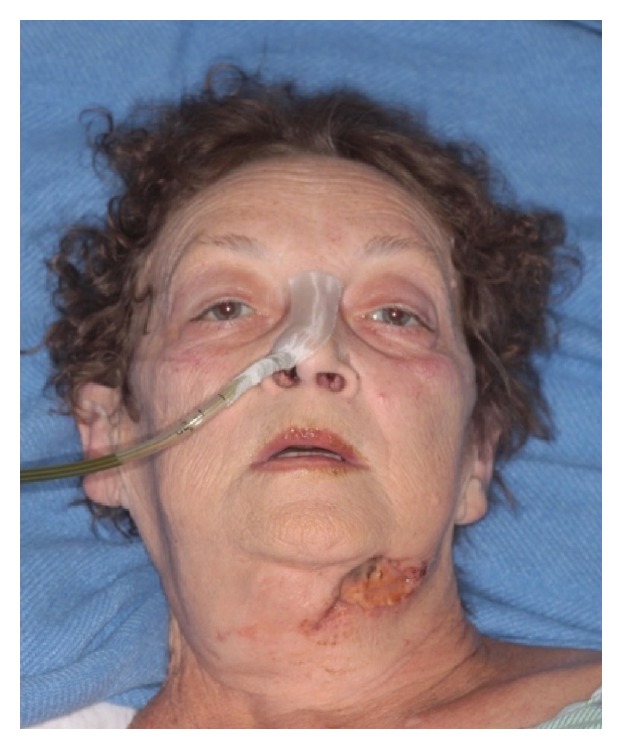
Patient presents to the emergency room in November 2013 with left submandibular fistula.

**Figure 2:**
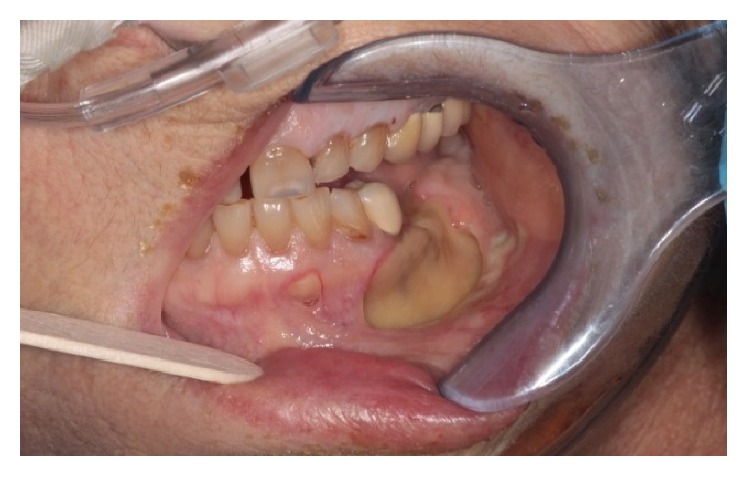
Intraoral photographs of necrotic, exposed bone surrounding site of extracted tooth #20.

**Figure 3:**
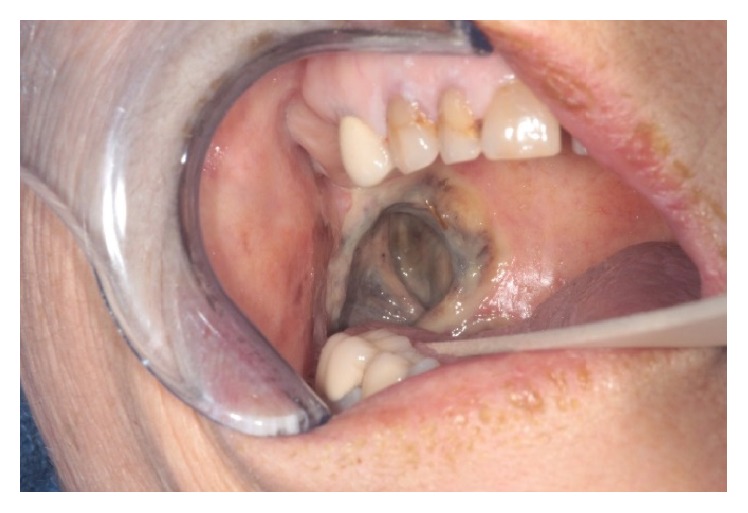
Intraoral photographs of soft tissue fistula in the right side of her soft palate.

**Figure 4:**
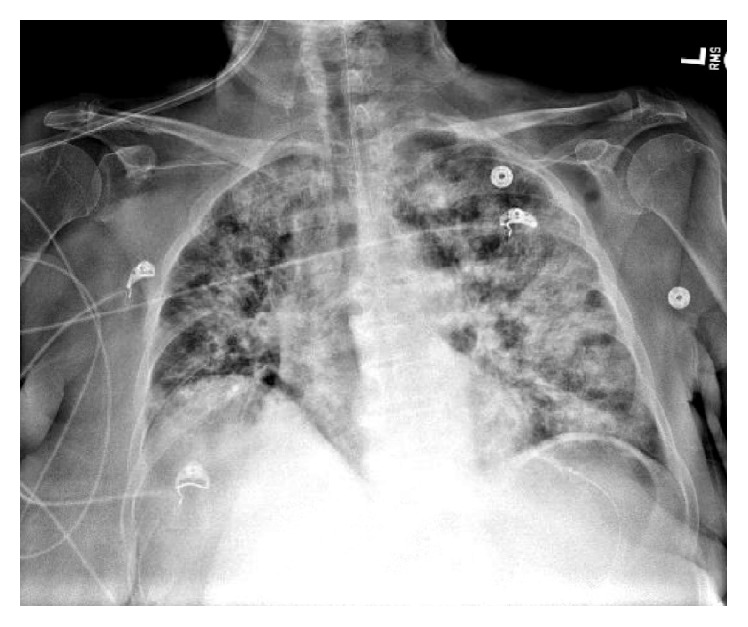
Chest X-ray demonstrating findings suspicious of ARDS.

**Figure 5:**
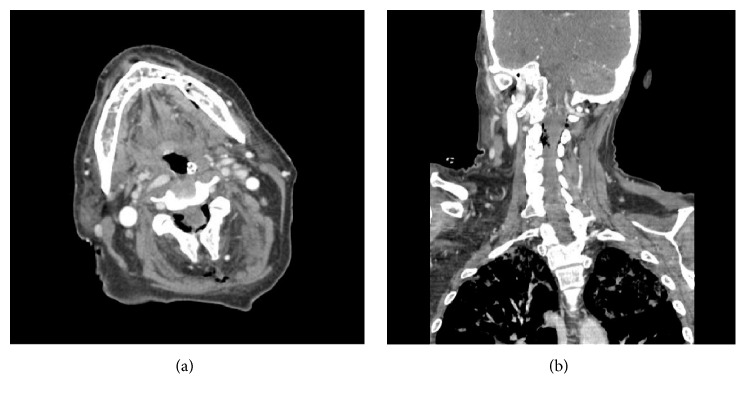
(a) CT scan shows no fluid collection or abscess. However, air is evident in the marrow on left side of the mandible. (b) CT scan showed air in the epidural space in cervical region.

A week later, the patient was transferred from an outside hospital to our intensive care unit with a diagnosis of sepsis. Her left neck fistula had doubled in size and now had a necrotic appearance. The inferior border of the mandible was visible in the wound (Figure 1). Intraorally, the area of bony exposure increased in size to 2.5–3 cm area in the left posterior mandible. The symphysis and right posterior mandible areas of bony exposure also increased in size (Figure 2). Moreover the right side of her soft palate appeared to have undergone spontaneous necrosis, forming a through and through 2 cm defect into the nasopharynx. This fistula did not communicate with any areas of bony necrosis on exam and endoscopy (Figure 3). The patient was started on broad spectrum antibiotics including Vancomycin, Levofloxacin, and Meropenem and ID was consulted. The patient was afebrile with a white cell count of 10.5 × 10^3^/mm^3^. She suffered from tachycardia and was tachypneic and her chest X-ray showed findings suspicious of Acute Respiratory Distress Syndrome (ARDS) (Figure 4). She required supplemental oxygen but no mechanical ventilation. She also received a blood transfusion at the outside hospital for hemoglobin of 5.0 g/dL and was transfused again in our facility for hemoglobin of 7.1 g/dL. She received multiple platelet transfusions for thrombocytopenia with a count 63 × 10^3^/mm^3^ on arrival and a nadir of 16 × 10^3^/mm^3^ (unit) during her 10-day hospital stay. The differential diagnosis by hematology was autoimmune causes due to her sepsis such as idiopathic thrombocytopenic purpura (ITP) versus drug related causes. The patient also had anion gap metabolic acidosis and was managed with fluids, insulin, dextrose, and bicarbonate and took several days to stabilize. Blood cultures eventually grew out* Escherichia coli* which was sensitive to the antibiotics administered. A repeat CT scan showed no abscess but showed some air in the marrow on left side of the mandible (Figure 5(a)). It also showed air in the epidural space in cervical region (Figure 5(b)). Neurosurgery recommended treatment with antibiotics.

As the patient progressed in her hospital course, she started to improve. Her anion gap was corrected, her counts improved, and her intra- and extraoral wounds stabilized. A PICC line was placed and the plan was to have the patient complete a 4-week course of Unasyn (Ampicillin/Sulbactam) after discharge followed by oral Augmentin (Amoxicillin/Clavulanate). Due to her generalized weakness and diffuse involvement of her mandible, the patient was deemed not a good surgical candidate at the time. There was also a concern with regard to soft tissue healing after surgery given her soft tissue wounds.

**Figure 6:**
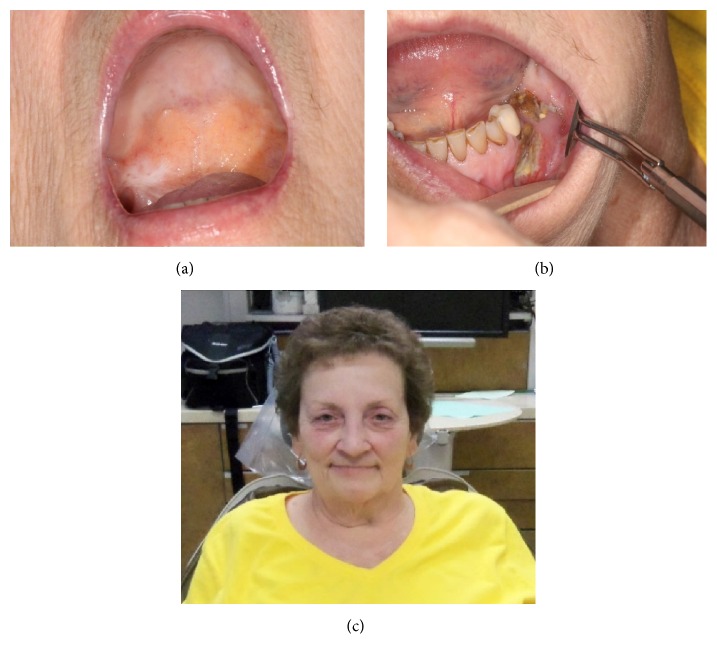
(a) Soft tissue healing intraoral wounds 1 year after initiation of MRONJ. (b) Improved bony coverage. (c) Healed neck fistula.

At follow-up 3 months after discharge, the palatal fistula was found to have completely healed. The neck wound had nearly completely healed with a pin point fistula with minimal drainage remaining. The 2 small areas of bony exposure in the symphysis and right posterior mandible had resolved. The left mandibular bony exposure was stable without progression. The patient continued to show progressive improvement on periodic follow-up. At 1 year, the soft palate and neck wounds were completely healed, and the intraoral wound had dramatically decreased in size with no purulence (Figure 6). At 18 months the patient continued to do well. She essentially had near complete recovery with no surgical intervention. The patient was content with area of exposed bone in her oral cavity and was not interested in any conservative surgical debridement.

## 3. Discussion

Denosumab is a competitive antagonist of the cytokine RANKL, which is integral to the differentiation and function of osteoclasts. Inhibition thus leads to decreased osteoclastic activity and bony turnover [15, 16]. Several phase III prospective randomized trials showed the superiority of Denosumab over BPs in preventing SREs in both osteoporotic and metastatic cancer patients [15, 17–20]. A meta-analysis, by Lipton et al., pooling the data from three randomized clinical trials with a total of 5723 patients showed a 17% reduction in SREs in the Denosumab arm versus zoledronate [4, 15, 21, 22].

Denosumab has a half-life of 25–32 days with its effects dissipating within 6 months of the cessation of treatment, as opposed to BPs which accumulate and persist in bone for years after stopping therapy [1, 4, 14, 21]. These pharmacokinetics help explain the spontaneous recovery of the lesions seen in our patient. Despite this, Denosumab has a higher incidence of ONJ with several clinical trials showing a 40% excess risk of developing ONJ in the Denosumab group as opposed to BPs [15, 19]. This is likely due to the increased efficacy of Denosumab in inhibiting osteoclasts.

In this case, the patient received 1 dose of Denosumab (Prolia) roughly a week prior to the extraction. She was on low dose bisphosphonates for treatment of osteoporosis for 4 years before switching over to Prolia. There are several plausible hypotheses that may help explain the presentation of the patient in this case report.

1. The fast onset and recovery of this case really favor Denosumab as the main culprit due to its short half-life in contrast to bisphosphonates. While one might assume that the soft tissue necrosis is due to antiangiogenic effects of medications, to date, Denosumab has not been shown to have antiangiogenic effects [23, 24]. However, RANKL is widely found in the body and affects many organ systems besides the skeletal system, including the skin and immune system [25, 26]. Immune cell precursors, as in the case of osteoclasts, require RANKL activation in order to differentiate and mature into active lymphocytes. This effect on immune cells and lymphopoiesis can predispose patients to infections. A study by Bridgeman et al. showed an 8 to 1 increase in the number of infections requiring hospitalizations in Denosumab patients compared to a placebo. Some of these infections included cellulitis, pneumonia, and sepsis, as seen in our patient. The combination of antiresorptive properties of the drug combined with the decreased ability to fight infections is the likely causes for the bony exposure and soft tissue necrosis seen in this patient.
2. Synergy between the BP and RANKL inhibitors may be another hypothesis to explain the presentation in this patient. Although the STAND trial suggests safety of taking these 2 medications in tandem for the treatment of osteoporosis [17], there are reports that suggest a higher incidence and severity of ONJ in patients taking both medications due to a synergistic effect [27]. Bisphosphonates have an antiangiogenic effect and can negatively affect the soft tissue healing therefore compounding the effect of RANKL inhibitors above.

The occurrence of Bisphosphonate and Denosumab ONJ is dose related and it occurs more frequently in patient's being treated for bony metastasis. Denosumab (Prolia) 60 mg subcutaneously every 6 months is used in the treatment of osteoporosis, while monthly injections with higher doses are used in the treatment of bony metastasis (Xgeva 120 mg) [1, 10]. The risk of MRONJ in patients being treated for osteoporosis is very low. MRONJ in patients being treated for cancer occurred 50 times more frequently than in patient with osteoporosis, with incidence rates of 0.7–1.9% and 0.04%, respectively. The rate of MRONJ in osteoporotic patients approached that of patients receiving a placebo [1]. Thus, with the data supporting a low risk of ONJ in these patients, the severity of this case with progressive soft tissue defects, bony exposure, and occurrence of life threatening sepsis was unusual.

Lastly, due to the fact that the patient was on two different antiresorptive medications, the above hypotheses cannot be validated with certainty. These theories are based on the available literature and our understanding of the pathophysiology of osteonecrosis of the jaw in patients on BPs and/or RANKL inhibitor and the effect of each on the soft tissues.

The aim of this case report was to present an unusual presentation not previously described in this subset of patients. It is important for physicians and oral healthcare providers to be familiar with these medications and be familiar with the potential side effects. A dental evaluation prior to starting such medications is warranted.
